# Exploring Inequalities in the Social, Spatial and Material Practices of Teaching and Learning in Pandemic Times

**DOI:** 10.1007/s42438-021-00267-z

**Published:** 2021-11-06

**Authors:** Jos Boys

**Affiliations:** Learning Environments Equality, Diversity and Inclusion Centre (LEEDIC), Faculty of Built Environment, University College London, The Bartlett, London, UK

**Keywords:** Learning environments, Equality, diversity and inclusion (EDI), Educational practices, Student engagement, Disability

## Abstract

This article conceptualises higher education as a complex and dynamic set of entangled social, spatial and material practices — enacted, adapted and contested across spaces and technologies as these interact with diverse learners, teachers, curricula and contexts. Using modes of enquiry that start from this inherent complexity and intersecting these with contemporary disability and education studies, I ask how some of the normative social and spatial practices of higher education are being surfaced by the pandemic. Rather than framing Covid-19 as a massive shift from ‘normal’ (face-to-face) to ‘abnormal’ (virtual) delivery modes, I propose that its impact both continues and alters assumptions about what constitutes ‘proper’ university education, and both perpetuates and disrupts what is ‘noticed’, valued and supported in conventional teaching and learning processes. To do this, I will focus on two themes in current HE practices in the UK, as examples of what such an approach can open-up to view. This starts from the already existing tensions, complexities and contradictions as to what should constitute appropriate teacher and student behaviours and settings, and how this ‘normality’ is often being perceived as being lost because of the pandemic. By engaging with existing literature about longer-term patterns of inequalities in access and inclusion across physical and virtual HE learning environments, I hope to show some underlying problems in how student competency is being evidenced in virtual as compared to physical space and some ways the pandemic has exposed the unevenness of diverse student and staff relationships to space, time and technologies and the differential impacts on their educational experiences.

## Introduction

The enforced pivot to remote learning across higher education worldwide since early 2020 because of Covid-19 has already been assessed by many stakeholder institutions, educational experts and researchers, as to its current impacts as well as longer-term implications (Hack 2020; Tinsley 2020; Marinoni et al. 2020; Wind and Quacquarelli 2021). In this article, I will consider this from a slightly different perspective, by exploring what the shift from predominantly co-presence to mainly online provision reveals about conventional educational practices. Rather than centring on the pandemic as a monumental change to ‘normal’ HE, I argue that it offers a valuable mechanism for investigating everyday common sense about what teaching and learning involves, who does it, what competencies are required and how these are evidenced in learning environments (whether physical or virtual). To explore these questions, the article will start from some already existing scholarship, pre-pandemic, that have challenged or complicated normative face-to-face teaching and learning practices, from both disability and education studies^1^. It will then focus on two aspects, to illuminate how such an approach might open-up useful fields of enquiry during and post-pandemic. These are, first, problems in how student competency is framed and recognised that tend to value particular kinds of behaviours and performances over others and, second, assumptions about how a ‘good’ academic or student manages their learning spaces, technologies and time that can (re)produce and perpetuate inequalities in access to, and inclusion in, higher education.

Overall, the aim is not to ignore the impact of the current large-scale shift from physical to online settings, but to go beyond normative unconscious biases that authentic teaching and learning practices are inherently face-to-face and that educational technologies are merely an ‘add-on’ — ‘understood as a supplement or addition, applied with the intention of “enhancing” existing pedagogical practices or learning experiences’ (Knox 2019: 358) — that can often be seen as imposed, alien and inferior. As with other postdigital scholarship, this paper starts from the understanding that learning technologies are no longer (in fact never were) a separate, specialist area of investigation from other aspects of teaching and learning. Rather, teaching and learning — across its conceptual, personal, social, material and virtual spaces — is (re)produced through inherently entangled spaces, humans, encounters, objects and technologies and need to be analysed not through isolated elements (even where these are then ‘added’ together) but as partial, complex and dynamic *practices*.^2^ In my own research, this has led to a focus on how diverse individuals make sense of, survive in, and learn through educational spaces, as the continuous negotiation of its everyday activities and settings. I suggest it is the very mundanity of such activity that allows it to go unnoticed and unremarked upon, when in fact it is actually *work* — what has been called ‘problematic accomplishments’ (Ryave and Schenkein 1974: 65). It takes time and effort to perform the everyday routines of higher education as obvious and natural and to re-adapt or ‘breach’ them (Garfinkel 1967: 37–38) whether as an academic or student. However, whilst particular sets of practices have often become solidified into ‘normal’ higher educational practices, these do not form neatly aligned coherent, comprehensive and stable frameworks, but often contain their own tensions and contradictions, as well as differences across courses, departments, institutions and countries. Exploring these processes thus requires what Geertz (1973) famously called a ‘thick description’; that is, it is a rich and layered account that accepts inconsistencies and does not result in a ‘solution’ or conclusion (Fenwick and Edwards 2010).

The work of ‘being’ an academic or student, then, is not obvious, straightforward or uncontested. Norms have to be learnt, or ignored, or challenged and changed, both by faculty and by learners, and through the multiple ongoing relationships between them and the institutional cultures, curricula and spaces in which they are located. How, then, can we open up to view how academic assumptions and ‘normal’ routines work to frame students’ competencies, actions and achievements in particular ways and not others? How can we unravel potential inequalities in students’ current access to, and experiences of, learning so as enable current pandemic-related shifts to inform processes for positive and more equitable educational change?

This approach does not provide any simple answers about how to improve teaching and learning. What it does is illuminate (Parlett and Hamilton 1972) some possible questions and modes of enquiry that can inform more detailed research about the enabling and disabling effects for various participants in specific learning situations, in order to inform future actions for individual faculty and students, as well as for HE institutions. In this article — based on secondary sources, inflected through personal experiences and other academics’ anecdotes — it has also been necessary to over-simplify and even stereotype ‘normal’ or conventional educational HE practices. Not all academics think that digital technologies are inferior or an ‘add-on’, and with the pandemic push to online learning, I have seen many colleagues actively embrace educational technologies as part of their teaching practice. But I have also seen the enforced removal of face-to-face teaching perceived as a terrible loss — the demise of something intangible, mysterious even — but also deeply vital to what counts as teaching and learning in higher education. This is despite the fact that for the majority of on-campus students, most of their study is already *not* done face-to-face. Learning with a teacher in both small group settings and large lectures makes up an increasingly small percentage of HE student experiences, with the majority of learning already taking place elsewhere — in libraries, informal learning hubs, cafes and at home — through student-led independent and collaborative modes of study. With the availability of video-recorded lectures, students were already staying away from on-campus lectures pre-pandemic. It is exactly these (already existing pre-pandemic) conditions that offer a good opportunity to reflect on our diverse and often inconsistent everyday assumptions about, and routines around, learning and teaching in HE as these are ‘breached’ by the impact of Covid-19. Now is a great time to surface ‘normal’ attitudes and everyday teaching and learning practices where these perpetuate educational inequalities. In higher education’s current disruptions of routine, and the discomforts and confusions of enforced and unexpected change, the everyday work of ‘doing learning — usually hidden — is revealed.

## Starting from a Different Angle

An immediate, and often unnoticed, response to the pandemic worldwide has been from disabled activists and disability studies scholars in academia and beyond. Long refused the choice to work or study at least partly remotely, many disabled people have noted how quickly governments and employers were able to shift to supporting non-face-to-face activities when it helped the able-bodied. With a partial return to face-to-face interactions, many disabled people (who have already been the most exposed by the pandemic) are again finding that their choices are being reduced as we return to ‘normal’ conditions and that accessibility and inclusion in online spaces throughout this period have anyway had complicated and uneven effects (Shakespeare et al. 2021; Brown et al. 2021; Puang 2021). This is underpinned by a strongly emerging literature on academic ableism and exclusionary spatial and material practices in higher education (Zhang et al 2010; Titchkosky 2011; Price 2011; Dolmage 2017; Kerschbaum et al. 2017;). As Dolmage says ‘the ethic of higher education still encourages students and teachers alike to accentuate ability, valorise perfection, and stigmatize anything that hints at intellectual (or physical) weakness’ (2017: 3). Including diverse disabled students and the learning (conceptual, social, material, pedagogic) spaces that suit them in higher education remains mainly an ‘add-on’ where people with impairments must continually prove themselves to be acceptable within conventional academic norms. Research and writing from this vitally important field will thus be my first set of sources to intersect with pandemic experiences, enabling a critical review of both what are assumed to be ‘proper’ student character traits, and how these are ‘seen’ and evaluated by teachers.

In addition, whilst responses to the pandemic have tended to take face-to-face teaching and learning as the norm — and from my experience across many HE institutions, subjects and levels, underpinned by a common-sense belief that online learning is inherently inferior to face-to-face — distance and virtual learning can now be considered fully mature as well as well-researched, having developed from correspondence education over at least 150 years (Anderson and Dron 2011). Academics and researchers in this field, as well as academic developers, instructional designers and educational technologists have long been promoting and delivering high quality remote, blended and hybrid forms of higher education. This not only has been paralleled by rather uneven evaluations of the resulting new forms of learning spaces (as reviewed for example by Temple 2007; Painter et al. 2013; Ellis and Goodyear 2016) but also includes some useful and nuanced comparisons of, and intersections between, online and face-to-face learning and teaching (Paechter and Maier 2010; Kemp and Grieve 2014; Paul and Jefferson 2019; Jones et al. 2021). Here, again, an immediate response to the online pivot was a kind of resigned frustration that it has taken so long, and that so many educators in higher education still struggle with, and resist, virtual, blended or hybrid learning methods^3^. Experiences and research that start from distance and online learning, then, will be my second set of sources for intersecting with pandemic times. Most crucially, these centre on what distance education research can teach us, not just about remote learning, but also about how students and teachers experience the already existing multitude of spaces in which they ‘do’ learning and teaching.

## (Re)doing the Everyday ‘Work’ of Higher Education

As I have discussed elsewhere (Boys 2016), the common sense assumptions and everyday routines that are the often unnoticed underpinning of teaching and learning practices are best revealed when these are breached — whether locally or externally as with a worldwide pandemic. As Sacks writes in ‘On Doing Being Ordinary’:

…one part of the job [doing being ordinary] is that you have to know what anybody/everybody is doing: doing ordinarily. Further, you have to have that available to do. (1984: 415)

Covid-19 has been a huge, unexpected breaching for many on-campus teachers and learners as they suddenly found that they no longer had it available to ‘do being ordinary’. The conventional disciplinary-inflected norms of weekly lectures and seminars (and/or lab or studio-based work) — and the unspoken rules of engagement that underpinned it — were decisively disrupted. At the same time, everyone was being asked to adapt to a different, online, learning environment. Most immediately — whilst this emergency situation did not pre-suppose a move at once to well-developed and effective online education (Hodges et al. 2020) — it did reveal many pre-existing poor teaching practices (Czerniewicz et al. 2020), as well as making visible how many academics struggled, in attempting to make the online space work as much as possible like face-to-face forms of delivery (rather than, for example, changing the shape of that delivery), in having limited understanding of educational technologies and in dealing with additional preparation workloads. The pandemic, then, painfully exposed what higher education teachers did not ‘know’ outside of normal routines. It also opened up to view the many differences in capacities and willingness to adapt at speed, as well as exposing the different (and differential) contexts in which academics work — from permanently tenured through to precarious and part-time labour and with very different domestic situations and demands.

These various ‘exposures’ intersected with academics being under pressure to act as normal as possible with students:

Collectively, it could be argued, lockdown asks us to create an illusion that the university is somehow still the entity it was, situated as it was. This is attempted by requiring the dispersed and sequestered bodies, many mired in confusion and contingency, to engage in an elaborate performance of wholeness, coherence, organisation, and professionalism via performances in front of the ‘portal’ of a screen. (Gourlay 2020: 809)

Doing the ‘ordinary’ work of higher education in this new situation, then, has forced academics to more consciously perform their various roles and to negotiate the tensions between and across the shock of change and the tendency (and sometimes requirement) to minimalise the effects of that change. At the same time, the work itself has become effortful and exhausting: it is no longer a known routine. This all too closely mimics the realities pre-pandemic for disabled faculty and students who have often had to do the extra (exhausting) everyday work of ‘passing’ as non-disabled and/or of negotiating the time-consuming processes of being given individual ‘reasonable adjustments’ and accommodations (Kerschbaum et al. 2017; Price et al. 2017; Price 2011, 2021). This is because ‘ordinary’ educational practices, pre-pandemic, already produced the problem of such involuntary breaching for many non-normative faculty and students — not just those with impairments but also around race, class, gender and sexuality — who have had to negotiate a place for themselves within and across higher education’s conventional norms. Disability studies scholar Rosemarie Garland Thomson calls this ‘misfitting’:

When the spatial and temporal context shifts, so does the fit, and with it meanings and consequences. (…) The discrepancy between body and world, between that which is expected and that which is, produces fits and misfits. The utility of the concept of misfit is that it definitively lodges injustice and discrimination in the materiality of the world more than in social attitudes or representational practices, even while it recognizes their mutually constituting entanglement. (2011: 593)

Making these complex discrepancies visible is an important step. By starting from Garland Thomson’s concepts of fitting and misfitting, we can also begin to investigate in detail the everyday misalignments ‘between body and world, between that which is expected and that which is’ in education to better understand the relational and situated intersections between people, encounters and learning spaces, and to unpick how differential and inequitable processes and experiences are perpetuated. Rather than locating disability (or other identity category) in a person as their problem to deal with, we need instead to critically examine relations between and across how abilities are named and judged as superior or inferior in higher education and how educational practices and settings both make solid and invisible (to ‘normal people’) particular discriminatory beliefs and processes.

For the rest of this article, then, I focus on just two aspects of how the pandemic-enforced shifting of higher education’s spatial and temporal contexts have reproduced or altered who fits and who misfits. I will first consider teachers’ perceptions of what ‘being’ a student entails, when shifted from mainly physical to mainly virtual spaces. What competencies are preferred and how are have these been recognised as ‘correct’ through previous face-to-face and now online performances? Second, I will explore academic and student experiences of negotiating the time, space and technologies of higher education, as a way to explore how normative assumptions ‘manage’ the complex and often problematic intersections between teaching and learning, and our diverse everyday lives.

## ‘Reading’ Learning Competencies in a Pandemic

Taylor and Shallish (2019) propose that assumptions about what constitutes learning and teaching competency and achievement are themselves racist and ableist and that these are embedded across the physical learning environment, everyday teaching practices and curriculum design, delivery and assessment. They call these assemblages ‘seemingly neutral practices in the academy because they construct able-bodiedness/mindedness as naturally occurring and empirically measurable’ (1). Of course, as they note, our individual (and institutional and social) understandings of what higher education is for are not straightforward, but complex, contested and often contradictory (Winkle-Wagner and Locks 2014). What, then, are the character traits of this stereotypical ‘competent’ student, and how do these become ‘readable’ by tutors? Taylor and Shallish suggest that there are already inherent tensions here. Students are framed, on the one hand, as already deserving high achievers since they now have a place at university, with this recognisable through, for example, confidence, focus, self-organisation and independence. On the other hand, students are assumed in need of support to become high achievers (either by providing educational and social opportunity and/or because student diversity is a good thing in its own right). One common term for framing these different understandings is student engagement, and as Kuh et al. (2007) note, its two critical features combine these different assumptions:

The first is the amount of time and effort students put into their studies and other educationally purposeful activities … The second component of student engagement is how the institution deploys its resources and organizes the curriculum, other learning opportunities, and support services to induce students to participate in activities that lead to the experiences and desired outcomes such as persistence, satisfaction, learning, and graduation. (44)

Just as different individuals, departments and institutions take a multiplicity of positions on what students ‘should be like’, they will interpret and evidence student engagement in a variety of ways. However, I suggest that lack of visible signs of paying attention (looking attentive, showing facial reactions, making direct eye contact) or of demonstrating an ability to join in (asking questions, having a point of view, confidently debating an issue) often leads to assumptions that a student may be unengaged, possibly gaming the system or just lacking a commitment to learning. Thus, who is valued and understood as deserving has often been most immediately ‘seen’ through the evidence of individual student performances in conditions of co-presence in the classroom. This is despite the fact that many students may not ‘present’ like this, for a diverse range of reasons that are not about lack of engagement, for example, because they have non-normative bodyminds (Price 2015) or because they are floundering in the gaps between what is implicitly expected but not explained and their own diverse backgrounds, knowledge and experiences.

With the pandemic, the data suggests that the most strongly felt frustration amongst academics is their inability to see students properly in an online environment, with demands for student’s cameras and microphones to be on at all times, despite acknowledging inequalities in students’ access to Wi-Fi bandwidth or spatial and aural privacy (Littlejohn et al. 2021). As well as the very real and unpleasant feeling of ‘talking into a void’ that teachers experience with not being able to see student faces, there is often a sense that students not making themselves visible undermines any possibility of engagement, or perhaps just as importantly, prevents teachers from garnering evidence of it. Whilst face-to-face lectures and other teaching contexts do not guarantee engagement — and in fact, students often fail to show exactly the kind of preferred visual cues and embodied actions suggested here in these settings too — being in physical proximity at least enables tutors to respond dynamically to their sense of what is going on in the room, and to feel they can make judgements about the authenticity and value of students’ engagement.

Here, whilst I note there are real issues of connection in online spaces, I want instead to draw out underlying assumptions about who is considered deserving of higher education and how this have always tended to act differentially, enabling some and disabling others. As many disability studies scholars and activists have shown, not showing ‘normal’ competency traits such as energy, independence, public-speaking confidence, quick processing or linear time management has been used historically to exclude disabled academics and students, as well as people of colour and other underrepresented groups from even entering higher education (Kerschbaum et al. 2017; Dolmage 2017). And for those who make it, not performing these ‘proper’ traits continues to affect how they will be viewed, often adversely (Ahmed 2012). For example, the assumed importance of actual attendance at lectures, pre-pandemic, has partly led to an ongoing resistance to making lecture recordings available afterwards or of flipping learning so that such content is made available either in advance of, or after, live taught sessions. Whether as ‘sage on the stage’ or ‘guide on the side’, there remains a tendency for academics to feel that co-presence with the teacher for formal elements of study (lecture, seminar, lab or studio sessions) is vital. This means that the values of processing time for learning, or for students to learn at different rates and in different ways — rather than competency only valorised through the performance of face-to-face, immediate, articulate responses — continues to be marginalised or ignored and not supported by curriculum design or delivery, whether in physical or online spaces.

## On Being Fair

If the shift to online learning and teaching has thrown into relief some of the anxieties and tensions about what makes a student deserving of higher education — literally because they often seem to disappear from view in online platforms — a related aspect is higher education’s attempts at ‘being fair’ in how assessment is undertaken, and achievements recognised, in pandemic conditions. In my own university, there has been an explicit ‘no detriment’ policy that has constructively aimed to take into account disruptions to student learning. As well as reviewing module and course marking schemes, this has also revisited the processes through which students request additional time for submissions because of extenuating circumstances (now extended to include the much larger numbers affected by Covid-19-related issues). Here, I suggest we can again see revealed individual, departmental and institutional (non-coherent) beliefs about how to recognise competency and to identify and separate out deserving from non-deserving cases. What kinds of ‘allowances’ should or are being made, who can be proven to deserve them and how do we assess ability in these difficult times? For Czerniewicz et al., this is not only centred around, but also complicated by, a strong academic commitment to students:

How we in education have attempted to ameliorate the challenges we and our students have encountered have taken the form of acts of care. Yet, every caring act occurs in a larger political context that reflects a given society’s values, laws, customs and institutions. (2020: 948)

This is not new. Struggles over how to formalise such care through explicit regulatory procedures and paperwork have been ongoing. Current HE institutional and departmental responses are underpinned by both policy-level and individual academic reactions to concepts such as reasonable adjustments and extenuating circumstances; this is in a context where cases have usually been framed as individual, to be proposed by the student themselves and then to be judged on the specific ‘merit’ of each case. What has changed is that potentially every student now enters the space of needing ‘accommodations’, previously reserved almost entirely for disabled and ‘problem’ students. This again reveals the limitations of the assumed high achiever learner stereotype, who is expected to always produce effectively and with continuing commitment, unimpeded by context or circumstances. Academic concerns then revolve around attempting to know — and police — who might be getting an ‘unfair advantage’ or gaming the system. As Taylor and Shallish (2019) note:

Individuals are understood to succeed because of their natural talents and hard work, rather than because they resemble the normed archetype of higher education fitness …

[and] accommodations are regarded as compensatory for deficits, thus positioning the university’s existing practices as ability neutral.

Even in pandemic times, students who struggle in juggling complex work-home-study situations, with managing their or relatives’ health conditions, or who need to apply for extra time for other reasons, are — in my experience — still often seen as threatening to the academic rigour assumed in conventional assessment methods, because of their perceived inability to perform as high achievers who can meet linear deadlines and conventional ‘progress’ narratives whatever their circumstances. Accommodations based on ‘objective’ assessments of individual extenuating circumstances may appear to provide a safety net for students, but often actually work to obscure more systematic time, space, technology and resource inequalities that already tend to enable some students and disable others. And this is not just about material inequalities (for example differential aspect to Wi-Fi and to study time). As Therborn writes, making students who need accommodations the problem, and leaving to them to apply for an alteration to the norm, also acts as a ‘denial of equal recognition and respect and is a potent source of humiliations’ (2020: 580). And, of course in addition, it makes the students, not the system, the stumbling block.

## Negotiating the Time, Space and Technologies of Learning

As well as attempting to judge students’ competencies at the level of direct appearances in classroom settings (face-to-face or virtually) against an assumed norm/ideal, then, this suggests that higher education simultaneously tends towards making other life competencies beyond the classroom and campus marginal or invisible. This leads to my second theme: how both staff and students negotiate the complex entanglements of their own lives with HE teaching and learning practices and how the pandemic has opened this up to critical view by shifting the locus from university spaces to online and domestic or other non-campus settings. This question must first be put in the context of wider assumptions about the added value of studying at a physical campus. Bayne et al.’s research suggests that even for distance students, the actual campus continues to be a ‘symbolically and materially significant mooring’ (2013: 581), although they also put this in the context that the very concept of distance education is assumed as a negative,

… [as] discursively determined and at the same time de-privileged via an explicitly spatial orientation which constitutes it as other to the ‘norm’ of the on-campus. (570)

They propose that this assumption of the campus as a superior ‘entity’ allows both its spatial presence and boundedness — separating insiders from outsiders — to literally make concrete (not just through physical space but also through, for example, promotional materials) the ‘special’ offer of an exclusive space for the kind of deserving high achievers already mentioned. At the same time, their study found that distance students, whilst needing some version of a physical campus to ground their studies also ‘relish(ed) their immersion in the networked … spaces of the online node’ (573). The authors argue that distance education students are able to hold positive notions of the campus as a real place and of the connectivity of their online course simultaneously, rather than view these as binary oppositions. With the pandemic, a much larger number of students and faculty are negotiating these kinds of symbolic as well as actual boundaries.

At the beginning of this article, I noted that students’ formal learning in teacher-led or facilitated settings is an increasingly small part of ‘normal’ campus-based studies. Many universities have been investing over several years in informal and peer group learning spaces, to provide spaces for students to study independently or collaboratively, partly in recognition of this fact. Unlike the class-based teaching and learning already explored, these individual and peer learning processes pre-pandemic do not assume academic co-presence for effectiveness. Instead, physical spaces take on a variety of roles from scholarly study in libraries through to more relaxed, collaborative and social situations in cafes and student hubs and to students’ own bedrooms and homes. How, then, is student (and staff) time, space and technology use being changed by Covid-19? What is the pandemic revealing about diverse and potentially differential and unequal experiences? Again, rather than assuming the pandemic as producing a dramatic shift to higher education practices, I will start with exploring how students negotiated the learning spaces available to them, pre-pandemic.

In 2013, Clare Melhuish and Angelina Wilson and I undertook a small pilot study of new undergraduate entrants to some courses at Northumbria University, UK. We wanted to find out how students entering university perceived and experienced their learning spaces, understood to include more than simply what was provided on campus (Boys et al. 2014). Rather than focusing on specific innovative learning spaces and on making and evaluating pedagogic and design improvements compared to existing ‘conventional’ provision — as is often the norm in learning environments research — we instead wanted to know how incoming students themselves negotiated the intersections between their lives, study, time and the spaces and technologies available to them. Used grounded theory and ethnographic research methods, through observations and focus groups of participants, complemented by photographic documentation from both students and researchers, we hoped to elicit meaning-laden and diverse responses from students’ perspectives. In particular, we were interested to see the effects of material settings within the inseparable entanglements among people, physical environments, learning technologies and educational practices.

The student photo diaries and focus group discussions showed the extent to which students learning took place on laptops in their own living spaces, including lying on their beds, using the bed as a desk and sitting in shared kitchens. Whilst we did gather data where our participants discussed the effects of their formal class sessions (across lecture, seminar, and studio spaces), the bigger concerns seemed to be more about what ‘being a student’ meant, what teachers’ expectations were and the anxieties and challenges this posed. This seemed most strongly felt in relation to developing skills in self-directed learning and thus was most intensely meshed with efforts to work out appropriate combinations of time, space and technologies for study, whether on campus, at home, or elsewhere. For our participants, only between 12 and 20 hours a week was in formal classes. In addition, they were juggling other aspects of their lives such as part-time employment or caring responsibilities. Becoming adequate (let alone high achieving) learners was thus quite directly a matter of coordinating effectively across and between personal time availability, course scheduling and travel distances/cost, accessing appropriate spaces and technologies for different learning activities and of dealing effectively with gaps between trying to understand and perform what ‘being a student’ entailed and the realities of their everyday lives.

The Bayne, Gallagher and Lamb (2013) study, already mentioned, was similar but of online distance students. As with our research, they started by eliciting ‘arrival stories’ (here with a Masters’ level cohort at Edinburgh University) as well as asking about study spaces and geographic locations. Their interviewees also revealed complex and nuanced relationships to each other, to their course and to institutional spaces, both in terms of practices and of the actual campus. As with our student subjects, they also used non-educational spaces for studying, this time of course to include peer and teacher facilitated online learning,

making their classroom in hotel rooms, offices, cafes, airports and buses, always via a bringing-together of multiple sociomaterial assemblages of laptops, dongles, internet connections, texts, teachers, and peers (Bayne et al. 2013: 580).

These suggest more complex educational shifts over the last year or two, not just a simplistic binary opposition between pre-pandemic face-to-face and post-pandemic online learning experiences. Of course, for the cohort of students caught up in pandemic restrictions who had expected to move to a university but in fact remained at home (or who did move and then found themselves in complicated situations in student accommodation), these kinds of multiple learning locations and technologies had to be negotiated without forward planning or an explicit choice in study method. For the academic year 2020–2021, I had students based in China who joined classes from a local shopping mall, or — off-camera — from corners of their family households or on a train. The key point here, however, is that there are continuities as well as disruptions in how students and staff have negotiated time, space and technologies before and during the pandemic and much to be learnt by intersecting pre-pandemic educational and disability studies research with current situations.

## Learning Time-space-tech in a Pandemic

Since the pandemic, the range of live-work-study options available to different students and teachers have of course become much more limited and have been further constrained by periods of socially distancing and self-isolation. The already existing pressures for many of studying in domestic spaces have been exacerbated as activities assumed kept apart (schooling, study, living, working, caring) have needed to be crammed into one location. But rather than framing this as a massive swing from one form of spatial relationship to another, we need to unravel the complex and differential effects on diverse participants across higher education and recognise the privilege that having access to space, time and material resources — such as good Wi-Fi and computing equipment, or a separate home study — continues to give to some and not others, even in these much more difficult circumstances (and to note how that such privilege often overlaps with, and thus reinforces, the particular backgrounds and experiences that help to generate the preferred character traits already mentioned).

There are now many studies of how diverse academics in the UK and internationally are managing their detailed arrangements when faced with this new situation (Gourlay et al. 2021; Littlejohn et al. 2021; Jandrić et al. 2020) as well as students’ views on their experiences (Higher Education Policy Institute 2020). As Gourlay writes, lockdown has required much creative improvisation as our domestic spaces are now not only about working/studying but also being on view to online audiences, requiring detailed re-arrangements of spaces and technologies:

Most of the participants describe an unfolding ‘making’ process, in order to create some work space. For some, this involved adding a work ‘layer’ to a space or set of artefacts also used for another purpose. In Lawrence’s case, he constructed his ramshackle structure of tables and boxes in order to be able to work comfortably. (Another participant’s ‘work space’ consisted of her sitting on her toddler’s rocking chair in the nursery room.) Mary used her sewing machine table as a stand for her laptop. Ellie transformed her bedside table into a miniature desk which was in fact too small to hold her laptop and notebook at the same time. Courtney rigged up a curtain to create a ‘professional’ background in her bedroom. (Gourlay 2020)

This has taken place in the wider context of ongoing differences in what is available to each person; in the various possible re-organisations and re-representations that, as already mentioned, straddle the tensions between ‘doing being ordinary’ on behalf of the university, or appearing as a ‘competent’ student; and admitting to or accidentally revealing not coping with workloads and/or everyday life. As Czerniewicz et al. (2020) note:

Students and staff were thrust into a lack of dedicated space to work undisturbed and the need to care for family members and especially children who must be home-schooled during the lockdown. Students reported more family responsibilities like running errands, household chores, taking care of elderly family members. Such role conflict emerged in stories of students being admonished for being lazy and just reading (rather than physically active); for having even more pressure to choose between prioritising their time/ finances for personal gain (their studies) or their families financial or care-giving needs. For some, returning home meant returning to places of violence while residential accommodation on campus was a refuge for those coming from abusive/dysfunctional homes—physical emotional and verbal abuse/gender-based violence.

In addition, with the sudden shift to entirely digital learning spaces, the impact on students and faculty has been various and complex. In the pivot to online learning, various ways of offering remote alternatives (break-out rooms, buddy groups, shared online whiteboards such as Padlet is or Miro) have been introduced, if somewhat unevenly. For disabled people, as Goggin says, the effects have cut in a variety of often contradictory ways:

…the tilt to digitalization has opened up or underwritten forms of inclusive participation for people with disabilities at the same time as new kinds of constraints and regulation of social life, freedom, and mobility have emerged. From many people with disabilities, we have often heard: ‘welcome to our world’; that is, now others have to participate in predominantly digital form across a much wider set of activities. Yet also many people with disabilities have been obliged to conform to the so-called ‘new normal’: teachers asked by schools and universities to switch to online, remote learning via platforms which have inadequate accessibility, poor affordances, and lack of inclusive design (2021: 2).

Critiques of time as an objective, regular and forward progress, being developed within disability studies, can also help in the understanding of different experiences of the pandemic for both students and academics (Samuels 2017; Price 2021). Time changed: routine things took extended time. For some people, time slowed and, for others, it sped up, whilst the boundaries between ‘work time’, ‘study time’ and ‘leisure time’ blurred. Again, the pandemic offers important research opportunities to bring such already existing differentiating experiences to the fore, to better understand how to improve equality, diversity and inclusion. By exploring both what students and teachers are ‘meant to be like’ and what sorts of spaces are ‘better’ for learning as these are struggled over because of the impact of Covid-19, we can begin to see how the time, space and technologies of higher education are already unevenly distributed and how these patterns are currently being both perpetuated and re-aligned.

## What Matters About Learning

In Jones et al.’s longitudinal study of Open University^4^ learners using a virtual design studio (VDS), involving 3000 students over 3 years, again what emerged

was a picture of student behaviour more complex and nuanced than originally expected. Far more emphasis seemed to be placed (by students) on the personal, psychological and social learning affordances in the virtual studio. (2021: 2)

Whilst the study monitored simple measurable actions in relation to achievement (coming online, viewing and adding content), a much stronger correlation was revealed between student success and the extent to which they viewed other students’ work. Counterintuitively — and compared to the sometimes obsessive focus in pandemic times for tutors to have students perform presence in virtual space — it was this more ‘passive’ behaviour that seemed to enhance learning, what has also been called ‘listening in’ (Cennamo and Brandt 2012) and ‘lurking’ where students are viewing/reading but not themselves contributing to online forums, online tutorials or discussion groups. As with research on language learning, and on peripheral learning as a first stage in becoming part of a community of practice (Lave and Wenger 1991), Jones et al. (2021) propose that it is these informal activities rather than the appearance of ‘paying attention’ that enhances students’ learning. It is such peer-peer behaviour that enables them to understand what is being asked of them, to gain confidence, to feel like they are doing the right thing in a particular learning context and to gauge their progress in relation to others.

For the researchers, there is a deeply important process of social comparison going on here — ‘Social learning mechanisms [that] are important in self-assessing personal capability.’ (Festinger 1954) the study then analyses which parts of the VDS software supported this kind of informal peer interaction and learning and which parts mitigated against it. They also explore the problem of maintaining social comparison throughout the course and of enabling students to make the shift towards being more self-directed learners who were more confident in their own judgements and are able to value and apply lessons from critical feedback as their studies progress.

Such concerns are just as central to teaching and learning in higher education more generally, whether face-to-face or online. I would suggest that it is through such social presence that students — separately and together — come to richly understand and negotiate ‘the rules of the game’ of higher education. This is not a matter of face-to-face ‘versus’ online learning; it is about developing high quality and equitable educational practices:

Using suitable learning design, students can be intrinsically motivated to use social comparison when viewing other students’ work. To achieve this, students use and develop social presence, even when they are engaging in less active behaviours, such as ‘listening-in’. As these become valuable, habitual actions, students are more likely to engage in further active engagements, which in turn, can lead to communities of practice emerging (Jones et al. 2021: 22).

Such an analysis is not isolated or unusual. From outside academia, many voices are also reminding us to value these intangible social qualities that we have missed during current times. This often stresses what is most important about co-presence:

The reason why offices are valuable is not because they uphold formal processes at work but what social scientists sometimes describe as ‘incidental information exchange’ (swapping ideas between teams) and ‘sense-making’ (navigating the world through shared knowledge and experience, including non-verbal cues Tett (2021: 54)).

But as Jones et al.’s (2021) study shows, such vital informal social interactions can also be effectively mirrored in online environments. If we are to learn from the extraordinary shifts higher education has had to make because of Covid-19, we need to be putting the pandemic years into this wider and longer-term educational and research context, to draw out — through proper evidence-based research — what really matters about both learning and the conceptual, material, virtual and social spaces in which it takes place.

## Towards More Equitable Teaching and Learning Practices

As I said in the ‘Introduction’, shifting mainstream higher education to online learning because of Covid-19 has happened in a hurry, with many academics and students very inexperienced in how to effectively ‘occupy’ virtual teaching and learning spaces, whilst often also operating in the most unconducive material circumstances. I called this a form of ‘breaching’ and have suggested that the pandemic is in fact a valuable mechanism for opening-up both assumptions about what constitutes a ‘proper’ university education and who is noticed, valued and supported in conventional teaching and learning processes. This is because previously unspoken and common sense educational norms and routines have been thrown into disarray and thus exposed to investigation and review. It is therefore a vital moment for critical reflection about our many inbuilt unconscious biases in higher education, across both individual positions and institutional cultures.

By exploring everyday educational practices as these are struggled over because of the impact of Covid-19, we can reach a deeper understanding of the contested spaces and gaps between what teachers may assume, what students are trying to understand about their learning context, what actually happens to enable effective learning and the impacts of different life experiences and situations. We can investigate more closely what counts as evidence of learning taking place and the role of virtual and/or physical environments in this process. We can begin to see how the encounters, space, time and technologies of higher education are already differentially distributed and how underlying discriminatory patterns are currently being ignored, challenged or reframed. Most crucially, by paying much more attention to what we assume the preferred character traits of a student (or academic) is or should be, we can open up to critical debate who gets noticed and valued in higher education and who is marginalised or made invisible.

For me, one of the more meaningful shifts generated by the pandemic has been the dramatic growth in academics connecting online in multiple ways, and through a variety of channels, to share ideas and discuss how to improve curricula, resources, modes of delivery and student experiences. Whether motivated by the difficulties of the challenges faced in moving to online and blended or hybrid methods and/or by recognising opportunities to rethink stereotypical norms and conventional educational practices, the emergence of these new spaces is one very positive outcome of Covid-19. Collaborative activities that are explicitly concerned with developing more equitable learning and teaching include Facknitz and Lorenz (2020) ‘Cripping Pandemic Learning in Higher Education’, Critical Design Lab’s ongoing ‘Accessible Teaching in the Time of Covid-19’ (2020) and Facebook groups like ‘Teaching in the Time of Corona: Resources’ (with 11,200 members) and ‘Online Art & Design Studio Instruction in the Age of Social Distancing’ (with 17, 400 members). Rather than just waiting for a time when higher education can ‘go back to normal’, then, the breaching effects of the pandemic are already generating new and innovative ways to collectively discuss and re-think everyday beliefs about how we learn and what we value.

## Data Availability

Data and material are all drawn from secondary sources.

## Footnotes

1 This article has not engaged with the important intersections and challenges between the two disciplines of disability and education studies. See, for example, Loutzenheiser and Erevelles (2019)

2 This approach, with many variations, has mainly developed across science and technology studies, actor-network theory, ethnomethodology, phenomenology, anthropology and geography (Garfinkel 1967; Sacks 1984; Callon 1987; Law and Hassard 1999; Thrift 2008; Ingold 2011). For a more detailed conceptual framework, see Boys (2016).

3 Personal conversation with Prof Diana Laurillard, Professor of Learning with Digital Techologies, UCL Knowledge Lab, a key figure in developing learning theories in the context of digital technologies since the 1990s.

4 It is worth noting that the OU educational model is open access, meaning that pre-requisite qualifications are not needed for enrolment on courses. This both requires modules to be designed for novice design students with diverse backgrounds and capabilities and to have an explicit focus in teaching and learning that values opportunities to offer paths toward social opportunity for students from diverse backgrounds.
